# Supplementary material for: Chr21 protein–protein interactions: enrichment in proteins involved in intellectual disability, autism, and late-onset Alzheimer’s disease
Source: Life Sci Alliance. 2022 Aug 1;5(12):e202101205. doi: 10.26508/lsa.202101205 (PMC9348576; doi:10.26508/lsa.202101205)
Supplement: Supplementary file 1 [file LSA-2021-01205_TableS1.docx]

TABLE S1

| **symbol** | **gene_id** | **locus** | **log2(foldchange)** | **p_value** |
| --- | --- | --- | --- | --- |
| Hrnr | XLOC_033132 | chr3:93319748-93333572 | -6,19996 | 5,00E-005 |
| Calm4 | XLOC_012657 | chr13:3837756-3838671 | -4,41869 | 5,00E-005 |
| Dsc1 | XLOC_025257 | chr18:20084702-20114773 | -3,75611 | 5,00E-005 |
| Dmkn | XLOC_046543 | chr7:30763755-30781066 | -3,29757 | 5,00E-005 |
| Krt6a | XLOC_019666 | chr15:101689927-101694305 | -3,03842 | 1,00E-004 |
| Krt1 | XLOC_019673 | chr15:101845425-101850786 | -2,94385 | 5,00E-005 |
| BC100530 | XLOC_021142 | chr16:36359381-36367570 | -2,88878 | 2,00E-004 |
| Stfa3 | XLOC_021144 | chr16:36450536-36455392 | -2,63572 | 5,00E-005 |
| Asprv1 | XLOC_043596 | chr6:86627851-86629704 | -2,37578 | 5,00E-005 |
| Krt7 | XLOC_018512 | chr15:101412402-101427806 | -2,36799 | 5,00E-005 |
| Krt13 | XLOC_009887 | chr11:100117331-100121495 | -2,31068 | 5,00E-005 |
| Col1a1 | XLOC_007904 | chr11:94936269-94953279 | -1,78837 | 5,00E-005 |
| Myl1 | XLOC_002337 | chr1:66924295-66945056 | -1,77732 | 5,00E-005 |
| Hba-a2 | XLOC_006854 | chr11:32296488-32297310 | -1,67528 | 5,00E-005 |
| Col1a2 | XLOC_042819 | chr6:4505696-4541543 | -1,62416 | 5,00E-005 |
| Hbb-bt | XLOC_049929 | chr7:103812523-103813923 | -1,58163 | 5,00E-005 |
| Dsp | XLOC_013070 | chr13:38151293-38198577 | -1,58158 | 5,00E-005 |
| Isg20 | XLOC_047329 | chr7:78913457-78920396 | -1,55536 | 1,00E-004 |
| Hba-a1 | XLOC_006852 | chr11:32283671-32284493 | -1,49398 | 5,00E-005 |
| Slc4a1 | XLOC_009958 | chr11:102348819-102365281 | -1,4836 | 5,00E-005 |
| Acta1 | XLOC_053806 | chr8:123891757-123894775 | -1,40555 | 5,00E-005 |
| Hbb-bs | XLOC_049930 | chr7:103826522-103827929 | -1,39015 | 5,00E-005 |
| Gypa | XLOC_051698 | chr8:80494044-80510785 | -1,35298 | 5,00E-005 |
| Alas2 | XLOC_058253 | chrX:150519519-150643860 | -1,31341 | 5,00E-005 |
| Fam46c | XLOC_034796 | chr3:100468061-100489192 | -1,27044 | 5,00E-005 |
| Slc25a37 | XLOC_017182 | chr14:69241850-69285103 | -1,26475 | 5,00E-005 |
| Hist1h4f | XLOC_014227 | chr13:23549826-23551643 | -1,23619 | 5,00E-005 |
| Car3 | XLOC_032527 | chr3:14863537-14872659 | -1,21121 | 5,00E-005 |
| Col14a1 | XLOC_017850 | chr15:55307749-55520931 | -1,16383 | 5,00E-005 |
| Hist1h3f | XLOC_012924 | chr13:23535417-23545054 | -1,14845 | 5,00E-005 |
| Ube2l6 | XLOC_028644 | chr2:84798827-84810003 | -1,12507 | 5,00E-005 |
| Col3a1 | XLOC_000367 | chr1:45311537-45349706 | -1,12386 | 5,00E-005 |
| Hist2h4 | XLOC_034710 | chr3:96260108-96263317 | -1,12032 | 5,00E-005 |
| Col6a2 | XLOC_005788 | chr10:76595755-76623404 | -1,11412 | 2,50E-004 |
| Col6a1 | XLOC_005789 | chr10:76708791-76726044 | -1,10701 | 5,00E-005 |
| Pecr | XLOC_002352 | chr1:72236969-72284314 | -1,09839 | 5,00E-005 |
| H19 | XLOC_050555 | chr7:142575530-142578146 | -1,09067 | 5,00E-005 |
| Lox | XLOC_025600 | chr18:52516059-52577325 | -1,08303 | 5,00E-005 |
| Rsad2 | XLOC_011621 | chr12:26442742-26456452 | -1,06633 | 5,00E-005 |
| Hist1h3a | XLOC_014238 | chr13:23761852-23762386 | -1,01477 | 2,00E-004 |
| Col6a3 | XLOC_002561 | chr1:90766859-90843971 | -0,985218 | 5,00E-005 |
| Rmrp | XLOC_037343 | chr4:43492784-43493059 | -0,942423 | 1,50E-004 |
| Hist4h4 | XLOC_045850 | chr6:136801552-136804431 | -0,892907 | 5,00E-005 |
| Hist1h1a | XLOC_012946 | chr13:23763667-23764414 | -0,829648 | 5,00E-005 |
| Igf2 | XLOC_050557 | chr7:142650767-142670356 | -0,802667 | 5,00E-005 |
| Lasp1 | XLOC_007996 | chr11:97798600-97799073 | -0,793349 | 1,50E-004 |
| Hist2h2bb | XLOC_033222 | chr3:96269699-96270192 | -0,732232 | 5,00E-005 |
| Postn | XLOC_032778 | chr3:54361106-54391041 | -0,700558 | 2,50E-004 |
| Snhg4 | XLOC_024615 | chr18:35553409-35558316 | -0,700372 | 1,00E-004 |
| Hist1h1d | XLOC_012927 | chr13:23555031-23558008 | -0,686673 | 5,00E-005 |
| Adcy1 | XLOC_006663 | chr11:7063488-7178505 | 0,601479 | 1,00E-004 |
| Nrip1 | XLOC_021488 | chr16:76285457-76373846 | 0,624407 | 1,50E-004 |
| Grm3 | XLOC_040886 | chr5:9485235-9726108 | 0,626478 | 5,00E-005 |
| Sla | XLOC_019032 | chr15:66670769-66850720 | 0,629759 | 5,00E-005 |
| Gucy1b3 | XLOC_034399 | chr3:82032003-82074711 | 0,636256 | 1,00E-004 |
| Syt7 | XLOC_026300 | chr19:10389089-10453346 | 0,643186 | 5,00E-005 |
| Kctd13 | XLOC_047941 | chr7:126928878-126945609 | 0,651424 | 2,50E-004 |
| Kcnj2 | XLOC_008295 | chr11:111066163-111076825 | 0,671585 | 1,00E-004 |
| Hs3st4 | XLOC_047891 | chr7:124358448-124398989 | 0,679226 | 5,00E-005 |
| Ube2ql1 | XLOC_014747 | chr13:69702831-69741027 | 0,689882 | 1,00E-004 |
| Pak6 | XLOC_029148 | chr2:118663576-118698020 | 0,695846 | 1,00E-004 |
| Necab1 | XLOC_037133 | chr4:14930640-15149131 | 0,6978 | 5,00E-005 |
| Caln1 | XLOC_040481 | chr5:130369457-130847599 | 0,718114 | 5,00E-005 |
| Gm26937 | XLOC_020441 | chr16:74957808-74971644 | 0,730074 | 5,00E-005 |
| Gm26307 | XLOC_020424 | chr16:74604103-74619741 | 0,815729 | 1,50E-004 |
| Ntsr1 | XLOC_030049 | chr2:180499975-180544979 | 0,819897 | 5,00E-005 |
| Dyrk1a | XLOC_020589 | chr16:94569995-94695519 | 0,820367 | 5,00E-005 |
| Nwd2 | XLOC_039687 | chr5:63646170-63810687 | 0,833642 | 2,00E-004 |
| Fam84a | XLOC_011507 | chr12:14130667-14152038 | 0,855347 | 5,00E-005 |
| Ptk2b | XLOC_017157 | chr14:66153256-66281052 | 0,878143 | 5,00E-005 |
| Drd1a | XLOC_014530 | chr13:54050024-54055658 | 0,921936 | 5,00E-005 |
| Pou3f2 | XLOC_037169 | chr4:22482094-22488366 | 0,92887 | 5,00E-005 |
| Gucy1a3 | XLOC_034400 | chr3:82092426-82216257 | 0,94632 | 5,00E-005 |
| Pou3f4 | XLOC_058047 | chrX:110790739-110814286 | 0,977451 | 5,00E-005 |
| Satb2 | XLOC_002232 | chr1:56793980-56971334 | 0,988086 | 5,00E-005 |
| Ccbe1 | XLOC_025738 | chr18:66047165-66319607 | 1,0228 | 5,00E-005 |
| Gm25578 | XLOC_001080 | chr1:124477815-124493530 | 1,13399 | 5,00E-005 |
| Mc4r | XLOC_025739 | chr18:66857704-66860487 | 1,20512 | 5,00E-005 |
| Xist | XLOC_059120 | chrX:103431516-103484957 | 1,4367 | 5,00E-005 |
| Fam25c | XLOC_016755 | chr14:34351881-34355393 | -inf | 5,00E-005 |
| Ly6d | XLOC_019072 | chr15:74762055-74763567 | -inf | 5,00E-005 |
| Lce1c | XLOC_033122 | chr3:92679246-92680918 | -inf | 5,00E-005 |
| Lce1a1 | XLOC_034583 | chr3:92646531-92648307 | -inf | 5,00E-005 |
| Lce1a2 | XLOC_034585 | chr3:92668612-92670315 | -inf | 5,00E-005 |

**Supplementary Table S1: Candidate genes from Whole-genome RNA sequencing of embryonic hippocampus from 189N3 DS mode**
