# Supplementary material for: Chr21 protein–protein interactions: enrichment in proteins involved in intellectual disability, autism, and late-onset Alzheimer’s disease
Source: Life Sci Alliance. 2022 Aug 1;5(12):e202101205. doi: 10.26508/lsa.202101205 (PMC9348576; doi:10.26508/lsa.202101205)
Supplement: Supplementary file 2 [file LSA-2021-01205_TableS2.docx]

TABLE S2

Chromosome 16 genes present in the triplicated region (Supplementary Figure S1 in Aziz et al., 2018) that are upregulated are indicated (yellow highlight).

| **symbol** | **gene_id** | **locus** | **log2(foldchange)** | **p_value** |
| --- | --- | --- | --- | --- |
| Fezf1 | XLOC_044453 | chr6:23245046-23248264 | -2,65041 | 5,00E-005 |
| Rxrg | XLOC_001471 | chr1:167598361-167639623 | -2,33322 | 5,00E-005 |
| Slc6a7 | XLOC_025672 | chr18:60995379-61014199 | -2,03635 | 5,00E-005 |
| Cpne7 | XLOC_052305 | chr8:123117373-123135185 | -1,98096 | 5,00E-005 |
| Dclk3 | XLOC_055247 | chr9:111439080-111489611 | -1,95633 | 5,00E-005 |
| Tmem132e | XLOC_007732 | chr11:82388899-82446327 | -1,95329 | 5,00E-005 |
| Pdyn | XLOC_031578 | chr2:129686548-129699938 | -1,93049 | 5,00E-005 |
| Gpr88 | XLOC_034957 | chr3:116249653-116253484 | -1,84376 | 5,00E-005 |
| Cpne6 | XLOC_015870 | chr14:55510447-55517431 | -1,73006 | 5,00E-005 |
| Fxyd7 | XLOC_048853 | chr7:31042514-31051454 | -1,6974 | 5,00E-005 |
| Rasal1 | XLOC_040309 | chr5:120648811-120680068 | -1,67329 | 5,00E-005 |
| Egr3 | XLOC_016057 | chr14:70077444-70082613 | -1,65988 | 5,00E-005 |
| Kndc1 | XLOC_048097 | chr7:139894695-139941540 | -1,65277 | 5,00E-005 |
| Cyp26b1 | XLOC_045219 | chr6:84571413-84593908 | -1,56419 | 5,00E-005 |
| Cdh22 | XLOC_032238 | chr2:165111506-165234737 | -1,49053 | 5,00E-005 |
| Nrgn | XLOC_055814 | chr9:37544492-37552745 | -1,49041 | 5,00E-005 |
| Epha8 | XLOC_038598 | chr4:136929418-136956816 | -1,45514 | 1,50E-004 |
| Tac1 | XLOC_042851 | chr6:7555070-7565835 | -1,42779 | 3,00E-004 |
| Strip2 | XLOC_042974 | chr6:29917012-29959680 | -1,40628 | 5,00E-005 |
| Barhl2 | XLOC_041884 | chr5:106452522-106458166 | -1,38455 | 2,50E-004 |
| Ikzf1 | XLOC_006677 | chr11:11686212-11772926 | -1,35448 | 5,00E-005 |
| Gng4 | XLOC_012721 | chr13:13784052-13827895 | -1,30694 | 5,00E-005 |
| Kcnf1 | XLOC_011523 | chr12:17172099-17176888 | -1,27384 | 5,00E-005 |
| Penk | XLOC_037045 | chr4:4133535-4188703 | -1,26797 | 5,00E-005 |
| Otof | XLOC_041253 | chr5:30367065-30461932 | -1,25292 | 1,50E-004 |
| Ngef | XLOC_002533 | chr1:87476828-87573870 | -1,24711 | 5,00E-005 |
| Lypd1 | XLOC_002748 | chr1:125676995-125913145 | -1,2412 | 4,50E-004 |
| AW551984 | XLOC_055843 | chr9:39587395-39617750 | -1,23729 | 5,00E-005 |
| Adora2a | XLOC_004210 | chr10:75316942-75334788 | -1,2167 | 1,00E-004 |
| 2010300C02Rik | XLOC_002052 | chr1:37611675-37719811 | -1,21195 | 5,00E-005 |
| Adamts15 | XLOC_055714 | chr9:30899154-30922452 | -1,19479 | 2,50E-004 |
| Kcnh1 | XLOC_001822 | chr1:192190871-192510158 | -1,18101 | 5,00E-005 |
| Cobl | XLOC_008677 | chr11:12236675-12464960 | -1,17534 | 3,00E-004 |
| Sorcs3 | XLOC_026810 | chr19:48206024-48805505 | -1,16221 | 5,00E-005 |
| Ace | XLOC_008227 | chr11:105965606-105989964 | -1,15276 | 5,00E-005 |
| Slc24a3 | XLOC_029462 | chr2:145168112-145641939 | -1,15216 | 5,00E-005 |
| Lars2 | XLOC_055422 | chr9:123366887-123462664 | -1,14679 | 5,00E-005 |
| Cpne5 | XLOC_023558 | chr17:29156520-29237790 | -1,12038 | 5,00E-005 |
| Hap1 | XLOC_009896 | chr11:100347326-100356141 | -1,11778 | 5,00E-005 |
| Rasgef1a | XLOC_043890 | chr6:118011539-118091546 | -1,11486 | 1,00E-004 |
| Neurl1a | XLOC_026802 | chr19:47178819-47259441 | -1,10916 | 1,00E-004 |
| C2cd4c | XLOC_005840 | chr10:79606853-79614025 | -1,0949 | 4,50E-004 |
| Tgfa | XLOC_043580 | chr6:86195250-86275449 | -1,08182 | 5,00E-005 |
| Pcdh1 | XLOC_025490 | chr18:38186346-38209762 | -1,06727 | 5,00E-005 |
| Dlgap3 | XLOC_036504 | chr4:127169270-127237218 | -1,0586 | 3,00E-004 |
| Grin3a | XLOC_037429 | chr4:49657015-49845769 | -1,05712 | 1,00E-004 |
| Trank1 | XLOC_055246 | chr9:111311738-111395775 | -1,02149 | 5,00E-005 |
| Gm26917 | XLOC_022519 | chr17:39842996-39848829 | -1,01927 | 5,00E-005 |
| Dnm1 | XLOC_030460 | chr2:32308337-32353304 | -1,01846 | 5,00E-005 |
| Htr2c | XLOC_058225 | chrX:146962437-147197277 | -1,01648 | 5,00E-005 |
| Car10 | XLOC_007885 | chr11:93098084-93601751 | -1,00962 | 1,50E-004 |
| Nell1 | XLOC_046842 | chr7:49975349-50863289 | -0,997497 | 5,00E-005 |
| Sst | XLOC_021007 | chr16:23889580-23890844 | -0,983389 | 1,50E-004 |
| Sertm1 | XLOC_034135 | chr3:54897068-54915887 | -0,976497 | 5,00E-005 |
| Pcdh11x | XLOC_058078 | chrX:120290326-120910618 | -0,930118 | 1,00E-004 |
| Ablim3 | XLOC_025690 | chr18:61799392-61911852 | -0,926076 | 5,00E-005 |
| Camk2a | XLOC_024873 | chr18:60925631-60988152 | -0,918257 | 1,50E-004 |
| Tmem130 | XLOC_042600 | chr5:144735914-144761578 | -0,902118 | 5,00E-005 |
| Gda | XLOC_027286 | chr19:21391306-21472661 | -0,898837 | 1,00E-004 |
| Nr2f2 | XLOC_049301 | chr7:70351906-70496351 | -0,898068 | 1,00E-004 |
| Pnmal2 | XLOC_046193 | chr7:16944681-16948828 | -0,866808 | 5,00E-005 |
| Zcchc12 | XLOC_057382 | chrX:36195903-36199158 | -0,836462 | 1,50E-004 |
| Eef1a2 | XLOC_032404 | chr2:181140895-181157844 | -0,814996 | 3,00E-004 |
| Cpne2 | XLOC_051884 | chr8:94533027-94570529 | -0,813148 | 4,50E-004 |
| Hpcal4 | XLOC_036416 | chr4:123183503-123194699 | -0,812715 | 2,50E-004 |
| Scg2 | XLOC_002438 | chr1:79434668-79440090 | -0,806703 | 1,00E-004 |
| Rph3a | XLOC_042168 | chr5:120940499-121010256 | -0,806569 | 3,00E-004 |
| Pvrl1 | XLOC_054322 | chr9:43744575-43807461 | -0,793971 | 3,50E-004 |
| Lppr4 | XLOC_034969 | chr3:117319145-117361038 | -0,785027 | 1,00E-004 |
| Vat1l | XLOC_052156 | chr8:114205639-114374070 | -0,779263 | 1,50E-004 |
| Atp1a3 | XLOC_048658 | chr7:24978168-25005895 | -0,738833 | 1,50E-004 |
| 1810041L15Rik | XLOC_019391 | chr15:84379202-84447097 | -0,721197 | 2,00E-004 |
| Nptxr | XLOC_019277 | chr15:79786350-79834333 | -0,718964 | 5,00E-005 |
| Clstn3 | XLOC_045628 | chr6:124430755-124464784 | -0,706838 | 2,50E-004 |
| Sirpa | XLOC_029332 | chr2:129592838-129632228 | -0,706525 | 4,50E-004 |
| Gabra5 | XLOC_049182 | chr7:57387271-57510059 | -0,692445 | 4,00E-004 |
| Dner | XLOC_002468 | chr1:84369838-84696221 | -0,683738 | 2,00E-004 |
| Kif11 | XLOC_026639 | chr19:37376402-37421859 | 0,639086 | 3,50E-004 |
| Atp5o | XLOC_021617 | chr16:91925222-91931630 | 0,646591 | 3,00E-004 |
| Cct8 | XLOC_021539 | chr16:87454984-87495869 | 0,673542 | 4,50E-004 |
| Dpy19l1 | XLOC_055677 | chr9:24411778-24503140 | 0,673796 | 3,00E-004 |
| Cenpe | XLOC_033592 | chr3:135212562-135273540 | 0,721894 | 5,00E-005 |
| Hspa13 | XLOC_021484 | chr16:75755190-75766818 | 0,726219 | 5,00E-005 |
| Nfe2l3 | XLOC_043244 | chr6:51432669-51458768 | 0,737098 | 2,00E-004 |
| Epha3 | XLOC_021454 | chr16:63532157-63864157 | 0,75295 | 1,00E-004 |
| Rai14 | XLOC_018634 | chr15:10568977-10714631 | 0,780773 | 1,00E-004 |
| Ndst3 | XLOC_035012 | chr3:123526165-123692193 | 0,783136 | 5,50E-004 |
| Ifngr2 | XLOC_020557 | chr16:91546884-91564007 | 0,788154 | 5,00E-005 |
| Cryzl1 | XLOC_021612 | chr16:91688897-91729103 | 0,789204 | 3,00E-004 |
| Sfrp1 | XLOC_050966 | chr8:23411501-23449632 | 0,793421 | 5,00E-005 |
| Spc25 | XLOC_030752 | chr2:69189998-69206190 | 0,816927 | 1,00E-004 |
| Satb2 | XLOC_002232 | chr1:56793980-56971334 | 0,816936 | 1,50E-004 |
| Dnajc28 | XLOC_021609 | chr16:91614256-91618999 | 0,843455 | 5,00E-005 |
| Xist | XLOC_059120 | chrX:103431516-103484957 | 0,853182 | 3,00E-004 |
| Robo2 | XLOC_021477 | chr16:74300204-74412416 | 0,877875 | 5,00E-005 |
| Slc25a13 | XLOC_044352 | chr6:6041217-6217173 | 0,88493 | 4,00E-004 |
| Sla | XLOC_019032 | chr15:66670769-66850720 | 0,903486 | 5,00E-005 |
| Tmem50b | XLOC_021608 | chr16:91574507-91597680 | 0,926597 | 5,00E-005 |
| Bmpr1b | XLOC_035120 | chr3:141837135-142169228 | 0,929287 | 1,50E-004 |
| Nrip1 | XLOC_021488 | chr16:76285457-76373846 | 0,934399 | 5,00E-005 |
| Gm19932 | XLOC_039565 | chr5:49396324-49431417 | 0,938757 | 5,00E-005 |
| Igf2 | XLOC_050557 | chr7:142650767-142670356 | 0,961835 | 5,00E-005 |
| Shisa2 | XLOC_015932 | chr14:59625280-59631660 | 0,972508 | 5,00E-005 |
| Postn | XLOC_032778 | chr3:54361106-54391041 | 0,975557 | 2,50E-004 |
| Snhg18 | XLOC_018766 | chr15:32240567-32244662 | 0,982755 | 4,50E-004 |
| Gm23441 | XLOC_033869 | chr3:17787460-17812829 | 1,01436 | 5,00E-005 |
| Bcl6 | XLOC_021009 | chr16:23965051-23988643 | 1,05294 | 2,00E-004 |
| 9130024F11Rik | XLOC_000448 | chr1:56971468-57042513 | 1,06453 | 5,00E-005 |
| Pou3f2 | XLOC_037169 | chr4:22482094-22488366 | 1,12585 | 5,00E-005 |
| Rorb | XLOC_027276 | chr19:18930608-19111196 | 1,14594 | 5,00E-005 |
| RP23-162G11.3 | XLOC_031352 | chr2:116066217-116085911 | 1,18192 | 5,00E-005 |
| Abracl | XLOC_005266 | chr10:18011259-18023411 | 1,24446 | 5,00E-005 |
| Fam46c | XLOC_034796 | chr3:100468061-100489192 | 1,25505 | 5,00E-005 |
| Hs3st3b1 | XLOC_009203 | chr11:63884692-63922284 | 1,26042 | 2,50E-004 |
| Thsd4 | XLOC_056159 | chr9:59966930-60546937 | 1,27541 | 5,00E-005 |
| Slc14a1 | XLOC_025952 | chr18:78100090-78142119 | 1,27925 | 1,50E-004 |
| Hba-a1 | XLOC_006852 | chr11:32283671-32284493 | 1,44257 | 5,00E-005 |
| Alas2 | XLOC_058253 | chrX:150519519-150643860 | 1,50173 | 5,00E-005 |
| Hba-a2 | XLOC_006854 | chr11:32296488-32297310 | 1,51777 | 5,00E-005 |
| Slc25a37 | XLOC_017182 | chr14:69241850-69285103 | 1,56786 | 5,00E-005 |
| Hbb-bs | XLOC_049930 | chr7:103826522-103827929 | 1,57062 | 5,00E-005 |
| Nrk | XLOC_058157 | chrX:138914429-139010482 | 1,5886 | 1,50E-004 |
| Gypa | XLOC_051698 | chr8:80494044-80510785 | 1,64558 | 5,00E-005 |
| 3110099E03Rik | XLOC_031341 | chr2:115493512-115514794 | 1,66241 | 2,00E-004 |
| Hbb-bt | XLOC_049929 | chr7:103812523-103813923 | 1,68489 | 5,00E-005 |
| H19 | XLOC_050555 | chr7:142575530-142578146 | 1,72107 | 5,00E-005 |
| 2610034M16Rik | XLOC_024075 | chr17:58878807-58991375 | 1,73634 | 4,00E-004 |
| Acta1 | XLOC_053806 | chr8:123891757-123894775 | 1,79686 | 1,00E-004 |
| Gm11892 | XLOC_035489 | chr4:24201377-24244569 | 1,80809 | 5,00E-005 |
| Gm11891 | XLOC_035509 | chr4:24385801-24430858 | 1,83672 | 5,00E-005 |
| Slc4a1 | XLOC_009958 | chr11:102348819-102365281 | 1,87194 | 5,00E-005 |
| Col1a2 | XLOC_042819 | chr6:4505696-4541543 | 1,89957 | 5,00E-005 |
| Hmga2 | XLOC_006415 | chr10:120361274-120477414 | 2,14441 | 5,00E-005 |
| Trim10 | XLOC_022473 | chr17:36869573-36877833 | 2,26393 | 5,00E-005 |
| Pou3f4 | XLOC_058047 | chrX:110790739-110814286 | 2,2692 | 5,00E-005 |
| Tnnt3 | XLOC_048182 | chr7:142460811-142516009 | 2,2982 | 1,50E-004 |
| Mylpf | XLOC_047954 | chr7:127208895-127214287 | 2,54322 | 5,00E-005 |
| Mc4r | XLOC_025739 | chr18:66857704-66860487 | 2,55312 | 5,00E-005 |
| Col1a1 | XLOC_007904 | chr11:94936269-94953279 | 2,64663 | 5,00E-005 |
| Ibsp | XLOC_039988 | chr5:104299286-104311472 | 2,87738 | 5,00E-005 |
| Hba-x | XLOC_006851 | chr11:32276599-32278115 | 3,39226 | 5,00E-005 |
| Hbb-y | XLOC_049933 | chr7:103851753-103853207 | 3,86809 | 5,00E-005 |

**Supplementary Table S2: Candidate genes from Whole-genome RNA sequencing of embryonic hippocampus from Dp(16)1Yey DS model**
