# Supplementary material for: Chr21 protein–protein interactions: enrichment in proteins involved in intellectual disability, autism, and late-onset Alzheimer’s disease
Source: Life Sci Alliance. 2022 Aug 1;5(12):e202101205. doi: 10.26508/lsa.202101205 (PMC9348576; doi:10.26508/lsa.202101205)
Supplement: Supplementary file 6 [file LSA-2021-01205_TableS6.docx]

TABLE S6

| **Gene** | **Reference** | **ICC Dilution** | **WB Dilution** |
| --- | --- | --- | --- |
| AGAP3 | Kindly given by Richard Huganir  (Johns Hopkins University) | 1:200 |  |
| APBA2 | Abcam ab105396 | 1:100 |  |
| BAF155 | Santa Cruz sc-10756 | 1:500 |  |
| BIN1 | Millipore 05-449 | 1:250 |  |
| CREBBP | Abcam ab50702 | 1:5 | 1:50 |
| CLU | Santa Cruz sc-8354 | 1:100 |  |
| DGCR8 | Proteintech 10996-1-ap |  | 1:500 |
| DLG1 | Santa Cruz sc-9961 | 1:20 |  |
| DLG2 | Neuromab clone N18:30 | 1:250 |  |
| DLG4 | Synaptic System 124-011 | 1:250 |  |
| DSCAM | Kindly given by Trosha Dwyer  (University of Toledo) | 1:2,000 | 1:1,000 |
| DYRK1A | Kindly given by Francisco Tejedor  (Instituto de Neurociencias CSIC-UMH) | 1:400 |  |
| DYRK1A | Cell Signaling #8765 |  | 1:800 |
| EP300 | Abcam ab3164 | 1:50 | 1:200 |
| E6TP1 | Santa Cruz sc-20846 | 1:50 |  |
| FAM53C | Santa Cruz sc-242750 | 1:200 |  |
| Fibrillarin | Cell Signaling #2639 | 1:400 |  |
| GFP | Abcam ab290 | 1:500 |  |
| GRIK1 | Santa Cruz sc-7617 | 1:100 |  |
| GRIN2AB | Synaptic System 244003 | 1:250 |  |
| HA | Covance clone 16B12 | 1:1,000 |  |
| HCN1 | Alomone APC-056 | 1:300 |  |
| HUNK | Santa Cruz sc-46144 | 1:300 |  |
| ITSN1 | Kindly given by Volker Haucke  (Leibniz-Institut für Molekulat Pharmakologie) | 1:200 |  |
| KLR | Millipore 07-122 | 1:500 |  |
| KCNJ6 | Alomone APC-006 | 1:500 |  |
| KCNQ2 | Alomone APC-050 | 1:200 |  |
| LIMK1 | Abcam ab39641 | 1:75 |  |
| MYC | Millipore 05-419 | 1:250 |  |
| RNASEN | Abcam ab85027 | 1:100 |  |
| RNASEN | Abcam ab1286 |  | 1:1,000 |
| SAPAP | Neuromab clone N127:31 | 1:100 |  |
| SMARCA2 | Abcam ab15597 | 1:500 |  |
| SMARCA4 | Santa Cruz sc-17796 | 1:500 |  |
| SNAP25 | Synaptic System 111011 | 1:500 |  |
| SPT7 | Kindly given by Makoto Kinoshita  (Nagoya University) | 1:100 |  |
| STX1A | Synaptic System 110111 | 1:500 |  |
| SYNPO | Synaptic System 163002 | 1:500 |  |
| TIAM1 | Santa Cruz sc-872 | 1:250 |  |
| TOMM20 | Santa Cruz sc-11021 | 1:100 |  |
|  |  |  |  |
| RIMS1 | BD Transduction Laboratories |  | 1:1,000 |
| RAB3 | BD Transduction Laboratories |  | 1:2,500 |
| SYN2A | BD Transduction Laboratories |  | 1:5,000 |
| MUNC13-1 | Synaptic Sytem |  | 1:2,000 |
| ACTIN | Santa Cruz |  | 1:1,000 |

**Supplementary Table S3: Antibodies dilution**

References and dilutions of the antibodies used as well as for *In situ* proximity ligation assays PLA and immunocytochemistry (ICC) studies and western blotting (WB) studies
